# WDR-5 exhibits H3K4 methylation-independent activity during embryonic development in C. elegans

**DOI:** 10.21203/rs.3.rs-7240678/v1

**Published:** 2025-08-04

**Authors:** Nurulhafizah Binti Samsudin, Kate Fisher, Gino B Poulin

**Affiliations:** University of Manchester; University of Manchester; University of Manchester

**Keywords:** Chromatin, H3K4 methylation, MLL/SET/COMPASS complex, chromosome X, WDR-5, RBBP-5, C. elegans embryo

## Abstract

**Background:**

Histone H3 lysine 4 methylation (H3K4me) is generally associated with active transcription and bivalent chromatin, but can also contribute to repression. In metazoans, H3K4 methylation is catalysed by KMT2 methyltransferases assembled with the core scaffolding proteins WDR5, ASH2L, and RBBP5. RBBP5 mediates complex assembly and nucleosome binding, whilst WDR5 stabilises interactions to promote tri-methylation. However, WDR5 also exhibits additional ‘moonlighting’ functions, leaving its specific roles in H3K4 methylation and transcription regulation unclear. Using *C. elegans* embryos, spike-in ChIP-seq, and null alleles of *wdr-5(-)* and *rbbp-5(-)*, we dissected the contributions of these scaffolds towards H3K4 mono-, di-, and tri-methylation as well as gene expression during *C. elegans* embryogenesis.

**Results:**

We show that *C. elegans* RBBP-5 is essential for both mono- and multi-methylated H3K4 deposition. On the other hand, WDR-5 is primarily required for H3K4me3, but can influence H3K4me2 and H3K4me1 deposition either positively or negatively depending on the genomic feature involved. We additionally performed RNA-seq on these mutants and found that *rbbp-5* deletion was largely tolerated with mis-regulation of ~ 700 genes, whereas the *wdr-5* deletion led to widespread transcriptomic disruption (~ 3000 genes). We initially hypothesised that these broad changes were driven by the altered H3K4me1 and H3K4me2 landscapes in the *wdr-5(-)* mutant. However, transcriptomic profiling of the *wdr-5(-)*; *rbbp-5(-)* double mutant, which lacks H3K4 methylation, revealed a high degree of similarity to the *wdr-5(-)* single mutant. This refuted our initial hypothesis and indicates that the changes in H3K4 methylation are unlikely to underlie the transcriptional effects of the wdr-5 deletion.

**Conclusions:**

Our findings strongly indicate that WDR-5 profoundly shapes gene expression through mechanisms beyond H3K4 methylation. Distinguishing between H3K4me-dependent and independent functions of WDR-5 will further understanding of its roles in development and disease.

## Background

The chromatin landscape is shaped by the organisation of nucleosomes within the confined space of the nucleus. The resulting epigenome facilitates the organisation of RNA polymerase II into transcription factories and regulatory hubs that help establish and maintain gene expression patterns during development [1]. Histones, in association with DNA form nucleosomes, the fundamental units of chromatin. Initially described as barriers to transcription, decades of chromatin research have since revealed that nucleosomes play far more nuanced roles, acting through post-translational modifications that can either promote or repress transcription [2, 3].

One of these modifications occurs on Histone 3 at Lysine 4 (H3K4me) and involves the covalent attachment of up to three methyl groups to form mono-, di-, or tri-methylated forms (H3K4me1, me2, and me3). These three modifications have distinct patterns of deposition and are broadly associated with transcriptionally active genes [4]. H3K4me3 is the most notable of these modifications as it is strongly correlated with active Transcription Start Sites (TSS) [5, 6]. H3K4me3 can regulate initiation and elongation during proximal-promoter pause-release of RNA-pol II [4, 7–9]. In addition to these roles, studies have shown that H3K4me2/me3 can generate bivalent domains when coupled with H3K27me2/me3. These bivalent domains regulate expression of developmental genes, which are initially repressed but poised to later resolve their transcriptional state [10]. In addition, H3K4me2 can also mediate non-coding RNA-mediated repression of Hox genes [11], whilst both H3K4me2 and H3K4me3 can suppress cryptic transcription [12–15]. Furthermore, enrichment in H3K4me1 together with H3K27 acetylation define transcriptionally active enhancers [16, 17]. Collectively, these studies suggest that alterations in H3K4 methylation may compromise transcriptional states by perturbing both activating and repressive mechanisms.

**Figure 1:**
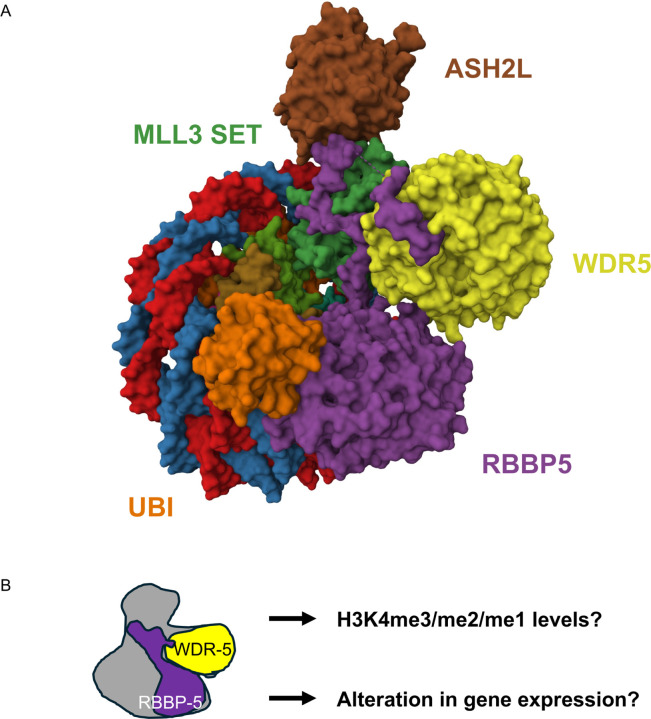
Cryo-electron microscopy structure of a representative SET/MLL complex with experimental rationale and description of the spike-in ChIP-seq approach. **(A)** The cryo-electron microscopy structure depicts the molecular surface of the core complex components WDR5, RBBP5, ASH2L, and the MLL3 catalytic module bound to nucleosome core particles containing mono-ubiquitinated H2BK120 (PDB: 6KIW; [21]). H2BK120ub1 acts as a trans-histone crosstalk signal to enhance H3K4 methylation. The complex adopts a Y-shaped conformation, with RBBP5 and WDR5 forming the arms. The complex rests flat on the nucleosome, contacting both DNA and core histones through RBBP5. RBBP5 anchors the complex to the nucleosome and physically interacts with DNA, histones, ubiquitin, and other complex components. In contrast, WDR5 bridges and organizes the MLL-RBBP5 interface. WDR5 can act as a context-dependent modulator, promoting H3K4 methylation in the MLL1 complex but acting repressively in the context of MLL3. **(B)** Schematic overview of the study objectives, which are to define i) the roles of RBBP-5 and WDR-5 in regulating H3K4 mono-, di-, and tri-methylation and ii) the downstream effects on gene expression, using spike-in ChIP-seq and RNA-seq approaches, respectively.

H3K4 methylation is catalysed by the evolutionarily conserved SET/COMPASS and MLL (Mixed Lineage Leukaemia) complexes [18–20] referred to hereafter collectively as the SET/MLL complex. These complexes consist of SET domain-containing methyltransferases (KMT2 enzymes) and a core scaffolding complex that together transfer methyl groups from the universal donor S-adenosylmethionine to H3K4, generating H3K4me1/me2/me3 (Fig. 1A;[21]). Notably, the number of KMT2 enzymes increases with species complexity: one in *S. cerevisiae*; two in *C. elegans*; three in *Drosophila*; and six in mammals [22, 23]. These enzymes form distinct SET/MLL sub-complexes that share core components but also include specific co-factors. The main core subunits are WDR5, RBBP5, ASH2L, and DPY30, which associate with co-factors such as CFP1 or Menin to form SET or MLL complexes, respectively [24]. Interestingly, WDR5 is also found in other chromatin-modifying complexes containing HAT or HDAC activities [25–32], challenging the attribution of WDR5-dependent transcriptional changes solely to its role in H3K4 methylation.

In *C. elegans*, the SET/MLL complex is comprised of two KMT2 enzymes: SET-2, a SET orthologue [33] and SET-16, an MLL-like enzyme [34]. The scaffolding subunits WDR-5, ASH-2, and RBBP-5 are highly conserved across species [33–36]. Independent loss of these scaffolding components alters H3K4 methylation and leads to phenotypes including altered lifespan [37–40], erroneous hindgut-to-neuron transdifferentiation [41], increased RAS signalling in vulval cells [34], axon guidance defects [42] and disrupted germ cell pluripotency [43, 44]. SET-16-dependent H3K4me3 also contributes to innate immunity [45]. Despite the clear roles of these components in H3K4 methylation, a comparative, quantitative analysis of their individual contributions has been lacking.

Herein, we determined the contribution of WDR-5 and RBBP-5 towards all three H3K4 methylation states using spike-in ChIP-seq on *C. elegans* embryos (Fig. 1B). We found that WDR-5 primarily prevents the deposition of tri-methyl groups at H3K4. In contrast, the absence of RBBP-5 abrogates all three states of H3K4 methylation. RNA-seq analysis show that *rbbp-5(-)* mutant embryos displayed fewer transcriptomic changes than *wdr-5(-)* embryos (721 versus 3377 Differentially Expressed Genes, respectively). Furthermore, the transcriptome of the *rbbp-5(-)*; *wdr-5(-)* double mutant, which lacks H3K4 methylation, closely resembled the *wdr-5(-)* single mutant. This indicates that gene expression changes in the *wdr-5(-)* mutant occur largely independently of H3K4 methylation. Together, our findings reveal that WDR-5 is required for H3K4me3 deposition and that it acts in parallel to H3K4 methylation to regulate gene expression.

## Materials and Methods

### Strains and general maintenance

*C. elegans* strains were maintained at 20°C and as described in Brenner et al. [46]. Strains used in this study are: N2 (wild type), *wdr-5.1(ok1417) III (RB1304), rbbp-5(tm3463) II*, and the double *rbbp-5(tm3463) II; wdr-5.1(ok1417) III* (OL76).

### Embryonic preparation on large solid media plates for spike-in ChIP-seq

Synchronised population of embryos were prepared on solid NGM media from bleached young adult hermaphrodites. These adults were harvested from two rounds of amplifications; each performed on 20 Corning tissue culture dishes (245 × 245 mm) that were seeded with synchronised L1 (larval stage 1) obtained from bleaching large numbers of gravid adults. To obtain these large populations of worms, dishes were seeded with 0.5 ml of 20x OP50 stock and left to dry for at least two days before use. 20 dishes per strain were seeded with synchronised L1s: ~1,000 L1s for N2 (wild type), ~ 2,000 L1s for *wdr-5(-)*, and ~ 3,000 L1s for *rbbp-5(-)*. When the next generation reached L4 stage, all worms were harvested and transferred onto 20 new dishes to prevent starvation and mature adults were bleached to generate large populations of synchronised L1s, which were all re-seeded onto 20 dishes. These L1s were grown to obtain young adults, a stage reached after 54h for N2 (wild type), and 64h for *wdr-5(-)* and *rbbp-5(-)*. Following bleaching of these adults, embryos were collected on sucrose gradients and re-suspended in 47 ml of M9 buffer and 2.8 ml of 37% formaldehyde solution (~ 2% final). Embryos were placed on a shaker (50 rpm) at room temperature for 30 minutes, then spun down and the pellets quenched by adding 50 ml of 100 mM Tris buffer (pH 7.5) to stop the fixation process. Fixed embryos were spun down and washed twice with 50 ml M9, then 10 ml FA buffer (50 mM HEPES/KOH pH 7.5, 1 mM EDTA pH 8, 1% Triton x-100, 0.1% sodium deoxycholate, 150 mM NaCl) containing protease inhibitor cocktail was added to the pellet. Between 200 μl and 500 μl of packed embryos can be obtained from this method. Next, an aliquot of 5 μl of embryos for each genotype was taken out, treated with methanol, and stained with DAPI for scoring on standard 2% agarose pad with a coverslip (store at −20°C). The embryo preparations used for N2 (wild type), *wdr-5(-)*, and *rbbp-5(-)* were comparable: 20–40% at < 28-cell stages; 20–27% between 28-cell and 100-cell stages; 32–52% at pre-coma stage; and 0–4% at coma stage or beyond. Then, samples were prepared as previously described [47]. Briefly, embryo pellets were re-suspended in a total of 1.5 ml FA buffer (50 mM HEPES/KOH pH 7.5, 1 mM EDTA pH 8, 1% Triton x-100, 0.1% sodium deoxycholate, 150 mM NaCl) with protease inhibitor cocktail. Embryos were dounced 40 times on ice using a pastel B homogeniser and aliquoted in 250 μl into six 1.5 ml microtubes. Sonication was performed using the Bioruptor UCD-200 (30 sec ON, 30 sec OFF) with ultrasonic wave output at HIGH for three 5 min cycles. Between cycles, the samples were cooled in a dry ice/ethanol bath for 5 sec. A small aliquot was kept to verify the DNA size on a 1.5% agarose gel showing enrichment between 400–600 bp. In the meantime, the samples were snap frozen and kept at −80°C. The frozen embryonic samples were then shipped to ActiveMotif on dry ice to perform the spike-in ChIP-seq procedure [48]. Briefly, about 5% of *D. melanogaster* was added to *C. elegans* embryo chromatin. 0.4 μg of the anti-H2Av antibody (AM39715) was used to immunoprecipitate *D. melanogaster* chromatin from each reaction, which is then used as a reference for normalisation. The antibodies are from ActiveMotif and are: anti-H3K4me1 (AM39297), anti-H3K4me2 (AM39141), and anti-H3K4me3 (AM39159).

### Spike-in ChIP-seq libraries and data processing

Illumina sequencing libraries were prepared from the ChIP and input DNAs by the standard consecutive enzymatic steps of end-polishing, dA-addition, and adaptor ligation. After a final PCR amplification step, the resulting DNA libraries were quantified and sequenced on Illumina’s NextSeq 500 (75 nt reads, single end).

All genomic analyses were performed using the *Caenorhabditis elegans* genome build WS220 (ce10). ChIP-seq reads were aligned to the ce10 genome using Bowtie2 v2.3.4 and processed with Samtools v1.9 and Picard. Duplicate reads were removed using MarkDuplicates, and mitochondrial reads were filtered out. To account for GC content bias, we applied computeGCBias and correctGCBias from DeepTools (v3.5.6) [49]. Normalisation by downsampling was carried out on the GC-corrected BAM files using Picard and spike-in normalisation factors applied. Final normalised coverage tracks (BigWigs) were generated from the downsampled, GC-corrected BAM files using bamCoverage with CPM normalisation and a bin size of 10 bp. These tracks were used in all downstream DeepTools analyses, including heatmaps, metagene plots, and principal component analysis.

Peak locations for overall genomic enrichment were determined using the MACS2 algorithm (v2.2.9.1) with an effective genome size of 9.3×107 and a q-value threshold of 0.05 [50]. For H3K4me2 and H3K4me3, broad peaks were called using default model-based parameters with a broad cutoff of 0.1. For H3K4me1, due to poor model estimation, peaks were called using ‘nomodel’ mode and a fixed fragment extension size of 147 bp. All peak calls were performed on GC-bias-corrected BAM files using matched input controls.

The N2 wild type data were benchmarked against modEncode publicly available early embryonic ChIP-seq dataset (H3K4me1 repl 1: GSM1217259 repl 2: GSM1217260 input: GSM1217261; H3K4me2 repl 1: GSM1206344 repl 2: GSM1206345 input: GSM1206346; H3K4me3 repl 1: GSM1206368 repl 2: GSM1206369 input: GSM1206370) [51].

To compare H3K4me1, H3K4me2, and H3K4me3 levels between wild type and mutants, Transcription Start Sites (TSS) generated by Chen et al. [52] and active enhancers mapped by Janes et al. [53] were used. Normalised bigWig files containing ChIP-seq signal coverage were analysed using Deeptools [49]. The bigWig files corresponding to wild-type (WT), *wdr-5(-)*, and *rbbp-5(-)* conditions were GC bias-corrected and normalised. BED files defining TSS and enhancer regions were uploaded for further processing.

### Embryonic preparations for RNA-seq

Synchronised populations of adult worms for N2, *rbbp-5(-)*, *wdr-5(-)* and *rbbp-5(-)*; *wdr-5(-)* were grown on 10 cm plates. Young adults were bleached and the embryos scored for developmental stages using DIC microscopy. The populations were consistently between ~ 25–30% at 28–100 cell stage, ~ 45–55% at pre-coma stage, and ~ 5–15% at late embryogenesis stages, roughly matching the spike-in ChIP-seq samples. Three biological replicates were prepared per genotype. Total RNA was extracted using TRIzol (Invitrogen) and stored at −80°C until sent for analysis to the Genomic core facility at the Faculty of Biology, Medicine and Health (University of Manchester).

### RNA-seq libraries and analysis

RNA-seq libraries were generated using the TruSeq Stranded mRNA Sample Preparation Kit (Illumina), followed by 101 × 101 bp paired-end sequencing on the Illumina HiSeq platform. Across 12 libraries, an average of ~ 208 million paired-end reads per sample were obtained (range: 100–422 million), with an average alignment rate of 94% to the *C. elegans* reference genome (ce10). Mate 1 and mate 2 reads were balanced across all samples, indicating high-quality libraries. FastQC and Trimmomatic [54] were used for quality control and adapter trimming. Reads were aligned using TopHat 2.1.0 [55], and gene-level quantification was performed using HTSeq [56] with the c_elegans WS220.annotations.gtf annotation file. Differentially expressed genes (DEGs) were identified using DESeq2 [57], applying thresholds of fold change > 2 and adjusted p-value < 0.05 with a cut-off for read counts set at 50 as detected in wild type and/or mutant samples.

### Calculation of Net Transcriptome Change Percent

To assess the global directional impact of transcriptomic changes in mutants relative to wild type, we calculated the Net Transcriptome Change Percent by integrating the direction of expression change (log_2_ fold change) with the level of gene expression (baseMean) for differentially expressed genes (DEGs). Only DEGs passing thresholds for both adjusted p-value and fold change were included in this analysis. For each mis-regulated gene, the baseMean (mean normalised expression across all samples) was multiplied by its log_2_-transformed fold change (log_2_FC). The contributions of all mis-regulated genes were summed to produce the Net Directional Change. The baseMean values for all DEGs were summed to calculate the Total BaseMean, representing the total transcript abundance for all mis-regulated genes irrespective of regulation direction. The Net Transcriptome Change Percent was calculated by expressing the Net Directional Change as a proportion of the Total BaseMean and multiplied by 100. This metric provides a global estimate of the directional bias in transcriptomic change, expressed as a percentage of the total mis-regulated gene expression. Negative percentages indicate net down-regulation; positive percentages indicate net up-regulation.

### Statistical test on the Violin plots

We tested whether genes classified as up-regulated, down-, or mis-regulated in a mutant background tend to have different expression levels **in wild type** compared to unregulated genes, using the baseMeanA metric (representing average wild-type expression). We performed a Mann-Whitney U test, also called a Pairwise Wilcoxon rank-sum tests, applied to log2-transformed baseMeanA values to assess distributional shifts across categories.

### ChIP-seq and RNA-seq data access

The ChIP-seq data have been deposited in the GEO repository under ID code GSE94639 and RNA-seq data have been deposited in the ArrayExpress repository under ID code E-MTAB-15080.

## Results

### Benchmarking H3K4 mono- and multi-methylation spike-in ChIP-seq data

**Figure 2:**
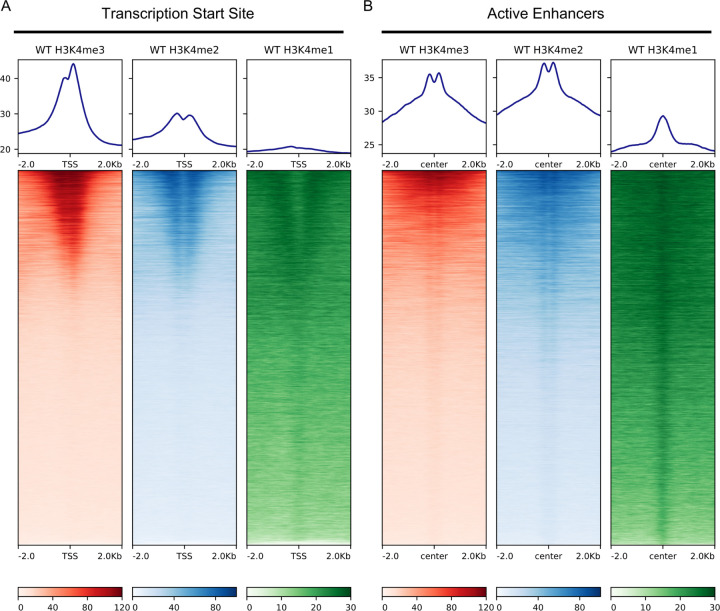
Spike-in ChIP-seq profiles reveal characteristic H3K4 methylation patterns at transcription start sites and active enhancers. **(A)** In wild-type *C. elegans*embryos, H3K4me3, H3K4me2, and H3K4me1 exhibit distinct enrichment patterns around transcription start sites (TSS), consistent with previous studies [47, 51, 53]. Aggregate plots (top) and heatmaps (bottom) show ChIP-seq signal for H3K4me3 (red), H3K4me2 (blue), and H3K4me1 (green) centred on TSS (±2 kb). **(B)** Enhancers in wild-type embryos also show distinct H3K4 methylation profiles. Aggregate plots (top) and heatmaps (bottom) show H3K4me3, H3K4me2, and H3K4me1 ChIP-seq signal centred on enhancer midpoints (±2 kb).

To assess the *in vivo* contributions of RBBP-5 and WDR-5 to H3K4 methylation, we performed spike-in ChIP-seq on *C. elegans* embryos for H3K4me1, H3K4me2, and H3K4me3. This approach enables accurate comparison across genotypes, especially important given the expected global reduction in H3K4 methylation in *rbbp-5(-)* and *wdr-5(-)* mutants. Spike-in normalisation is critical for such cross-genotype comparisons in ChIP-seq [58]. In our study, we used *Drosophila* chromatin and an antibody against the *Drosophila*-specific histone variant H2Av to normalise for technical variability during the ChIP process [48]. Normalisation analysis confirmed its necessity, particularly in the *rbbp-5(-)* background for all three marks (H3K4me3/me2/me1; Suppl. Figure 1). In the *wdr-5(-)* mutant, the normalisation also produced a reduction in the number of usable tags, but to a lesser extent than in the *rbbp-5(-)* mutant. By contrast, the wild type samples were either unaffected (H3K4me3/me2) or only marginally affected (H3K4me1) by normalisation (Suppl. Figure 1). To benchmark data quality, we compared our spike-in ChIP-seq datasets with publicly available modENCODE H3K4me1/2/3 data [47, 51]. Principal Component Analysis (PCA) demonstrated clustering of our samples alongside modENCODE replicates according to H3K4 methylation state (Suppl. Figure 2A), confirming reproducibility. Pearson correlation analysis further validated high concordance between our data and the modENCODE datasets (Suppl. Figure 2B). Finally, we assessed the genomic distribution of H3K4 methylation at transcription start sites (TSS) of protein-coding genes and at active enhancers [52, 53]. As expected, H3K4me3 was enriched at TSS (Fig. 2A), whilst H3K4me1 showed characteristic enhancer enrichment (Fig. 2B), consistent with published data [47, 53]. Together, these analyses confirm that our spike-in ChIP-seq data are robust, well-normalised, and comparable to established datasets.

### RBBP-5 is required for bulk H3K4 methylation, whilst WDR-5 is critical for H3K4me3

**Figure 3:**
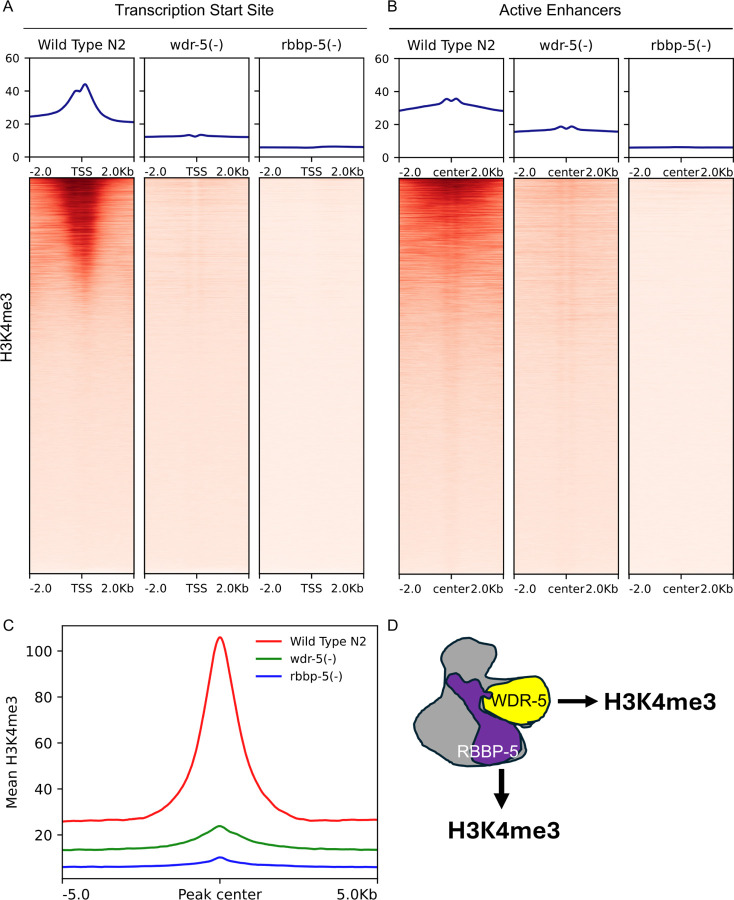
*RBBP-5 is required for bulk H3K4 methylation, whilst WDR-5 is critical for H3K4me3 deposition*. (**A and B**) Heatmaps showing H3K4me3 ChIP-seq signal centred on transcription start sites (TSS) and enhancer midpoints (±2 kb) in wild-type (N2), ***wdr-5(-)***, and ***rbbp-5(-)*** embryos. Both WDR-5 and RBBP-5 are critical for H3K4me3 deposition. (C) Aggregate plots show mean ChIP-seq signal centred on H3K4me3 peaks (±5 kb) in wild-type (N2), ***wdr-5(-)***, and ***rbbp-5(-)*** embryos. **Global** H3K4me3 enrichment is reduced in both ***wdr-5(-)***, and ***rbbp-5(-)*** mutants compared to wild type. **(D)** Schematic depiction of the WDR-5 and RBBP-5 positive contributions to H3K4me3.

To establish the contribution to H3K4 methylation from these scaffolding components, we compared the deposition of H3K4 mono- and multi-methylation between wild-type (N2) and the *wdr-5(-)* and *rbbp-5(-)* mutants. We first analysed H3K4me3 enrichment. In the absence of RBBP-5, H3K4me3 was almost completely lost at both TSS and enhancers (Fig. 3A and B). In *wdr-5(-)* embryos, H3K4me3 levels were also markedly reduced at TSS and enhancers, although the depletion was less severe than in the *rbbp-5(-)* mutant (Fig. 3A and B). Global H3K4me3 levels mirrored these findings, with the most pronounced reduction observed in the absence of RBBP-5 (Fig. 3C). We also constructed a schematic model to illustrate the relative contributions of RBBP-5 and WDR-5 (Fig. 3D).

**Figure 4:**
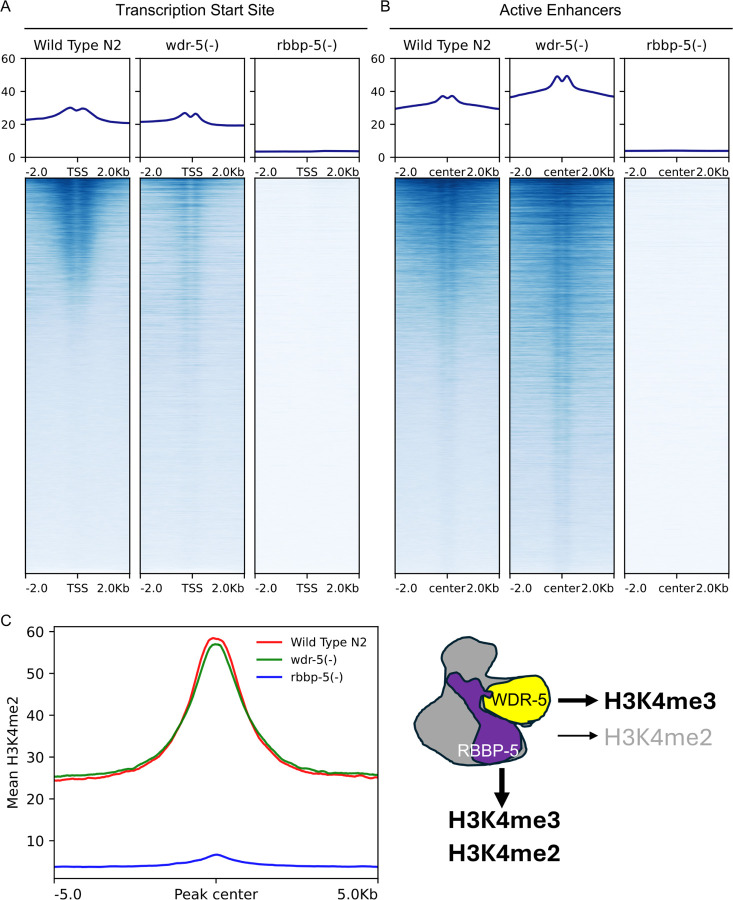
*WDR-5 modulates H3K4me2 levels in a genomic feature-dependent manner*. (**A and B**) Heatmaps showing H3K4me2 ChIP-seq signal centred on TSS and enhancer midpoints (±2 kb) in wild-type (N2), ***wdr-5(-)***, and ***rbbp-5(-)*** embryos. WDR-5 contributes positively to H3K4me2 deposition at TSS but negatively at enhancers. In contrast, RBBP-5 is critical for H3K4me2 enrichment. (C) Aggregate plots show mean ChIP-seq signal centred on H3K4me3 peaks (±5 kb) in wild-type (N2), ***wdr-5(-)***, and ***rbbp-5(-)*** embryos. **Global** H3K4me2 enrichment is not affected in ***wdr-5(-)*** compared to wild type whereas it is heavily depleted in the ***rbbp-5(-)*** mutant. **(D)** Schematic depiction of the RBBP-5 positive contributions to H3K4me3 and H3K4me2. WDR-5 contributes positively to H3K4me3 and exhibits positive and negative modulatory activities towards H3K4me2.

We next examined H3K4me2 deposition. As with H3K4me3, the absence of RBBP-5 resulted in an almost complete loss of H3K4me2 enrichment at both TSS and enhancers (Fig. 4A and B). In contrast, the effects of WDR-5 loss on H3K4me2 were context-dependent: H3K4me2 levels were reduced at TSS, but increased at enhancers (Fig. 4A and B). These opposing changes appeared to balance each other, resulting in no significant difference in the global H3K4me2 levels (Fig. 4C). We incorporated these context-specific effects into our schematic model to reflect the nuanced contribution of WDR-5 to H3K4me2 deposition (Fig. 4D).

**Figure 5:**
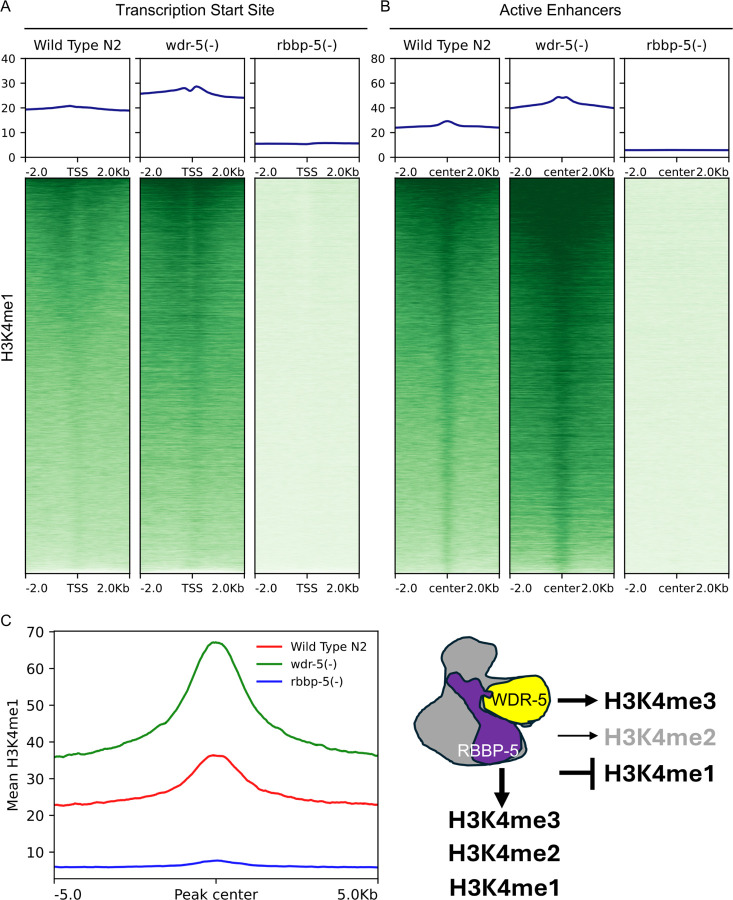
WDR-5 negatively impacts on H3K4me1. (**A and B**) Heatmaps showing H3K4me1 ChIP-seq signal centred on TSS and enhancer midpoints (±2 kb) in wild-type (N2), ***wdr-5(-)***, and ***rbbp-5(-)*** embryos. WDR-5 impacts negatively on to H3K4me1 levels at both TSS and enhancers. In contrast, RBBP-5 is critical for H3K4me1 enrichment. **(C)** Aggregate plots show mean ChIP-seq signal centred on H3K4me1 peaks (±5 kb) in wild-type (N2), ***wdr-5(-)***,and ***rbbp-5(-)*** embryos. **Global** H3K4me1 enrichment is increased in ***wdr-5(-)*** compared to wild type whereas it is heavily depleted in the ***rbbp-5(-)*** mutant. **(D)** Schematic depiction of the RBBP-5 positive contributions to H3K4me3, H3K4me2 and H3K4me1. WDR-5 contributes positively to H3K4me3, exhibits positive and negative modulatory activities towards H3K4me2, and impact negatively on the H3K4me1 levels.

Finally, we analysed H3K4me1 levels. RBBP-5 was essential for H3K4me1 deposition at both TSS and enhancers (Fig. 5A and B), a requirement that also held true at the global level (Fig. 5C). In contrast, the absence of WDR-5 led to an increase in H3K4me1 at TSS, enhancers, and globally (Fig. 5A–C). This accumulation may result from impaired progression to higher methylation states, leading to the build-up of H3K4me1. These data were integrated into our schematic to illustrate the distinct roles of RBBP-5 and WDR-5 in regulating H3K4me1 (Fig. 5D). Overall, these results show that RBBP-5 is essential for all H3K4 methylation states, whereas WDR-5 is primarily required for H3K4me3. These findings highlight the distinct roles of these scaffolding components in shaping the H3K4 methylation landscape.

### Chromosome X is differentially impacted by the absence of WDR-5 relative to autosomes

**Figure 6:**
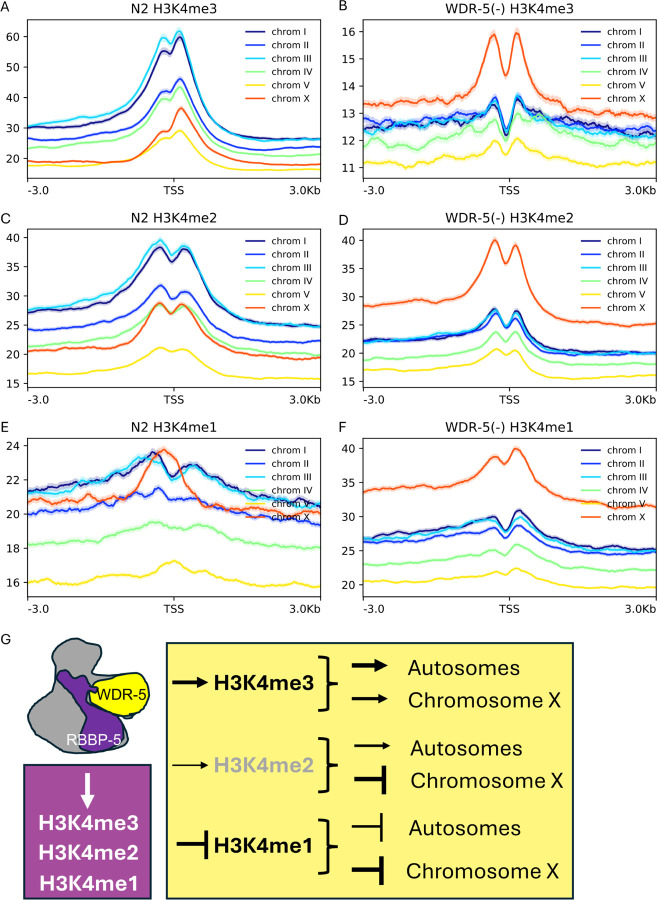
Chromosomal distribution of H3K4 methylation is differentially affected in the absence of WDR-5. Aggregate ChIP-seq signal plots show H3K4 methylation (±3 kb from TSS) for each chromosome in wild-type (N2) or ***wdr-5(-)*** embryos as indicated. (**A and B**) H3K4me3 levels at TSS are strongly reduced on autosomes in *wdr-5(-)* embryos but are less affected on the X chromosome. (**C and D**) H3K4me2 levels are mildly reduced on autosomes but increased on the X chromosome in the absence of WDR-5. (**E and F**) H3K4me1 levels increase for all chromosomes in *wdr-5(-)* embryos, with a more pronounced gain on the X chromosome. **(G)** Summary model illustrating the chromatin regulatory roles of RBBP-5 and WDR-5 in H3K4 methylation. RBBP-5 is required for deposition of all three methylation states. WDR-5 contributes positively to H3K4me3 deposition across the genome, promotes H3K4me2 on autosomes but represses it on the X chromosome, and restrains H3K4me1, especially on the X chromosome.

To address whether the absence of WDR-5 affects each chromosome similarly, we analysed H3K4 methylation enrichment at TSS for each chromosome individually. In wild type, the chromosomes with highest levels of H3K4me3 levels were chromosomes I and III, whilst chromosomes II and IV displayed intermediate levels, and chromosomes X and V showed the lowest levels (Fig. 6A). We examined how this chromosomal hierarchy of H3K4me3 levels was affected in the *wdr-5(-)* mutant. As expected, we found a striking reduction, but chromosome X was not as profoundly affected (Fig. 6B). We next analysed H3K4me2 levels and found that chromosomes clustered similarly to the H3K4me3 analysis with high and intermediate levels still being represented by chromosome I and III and chromosome II and IV, respectively. However, relative H3K4me2 levels increased on chromosome X (Fig. 6C). Interestingly, in the absence of WDR-5, autosomes exhibited a reduction in H3K4me2 enrichment, but the chromosome X displayed a robust increase (Fig. 6D). We then analysed the levels of H3K4me1, and found that in wild type, chromosome X was among the chromosomes with the highest and most characteristic H3K4me1 enrichment (Fig. 6E). As expected, in the *wdr-5(-)* deletion, all chromosomes displayed an increase in the levels of H3K4me1, but the chromosome X was the most affected (Fig. 6F). Together these findings reveal that while WDR-5 is broadly required for H3K4 multi-methylation across the autosomes, chromosome X appears to be subject to a WDR-5-independent regulatory mechanism that sustains or even enhances H3K4 multi-methylation in its absence (Fig. 6G).

### WDR-5 has a greater impact on the transcriptome than RBBP-5

Given the distinct effects of WDR-5 and RBBP-5 on H3K4 methylation, we next asked how these differences would be reflected at the transcriptome level. To address this, we performed RNA-seq on staged embryos and identified differentially expressed genes (DEG) relative to wild type.

**Figure 7:**
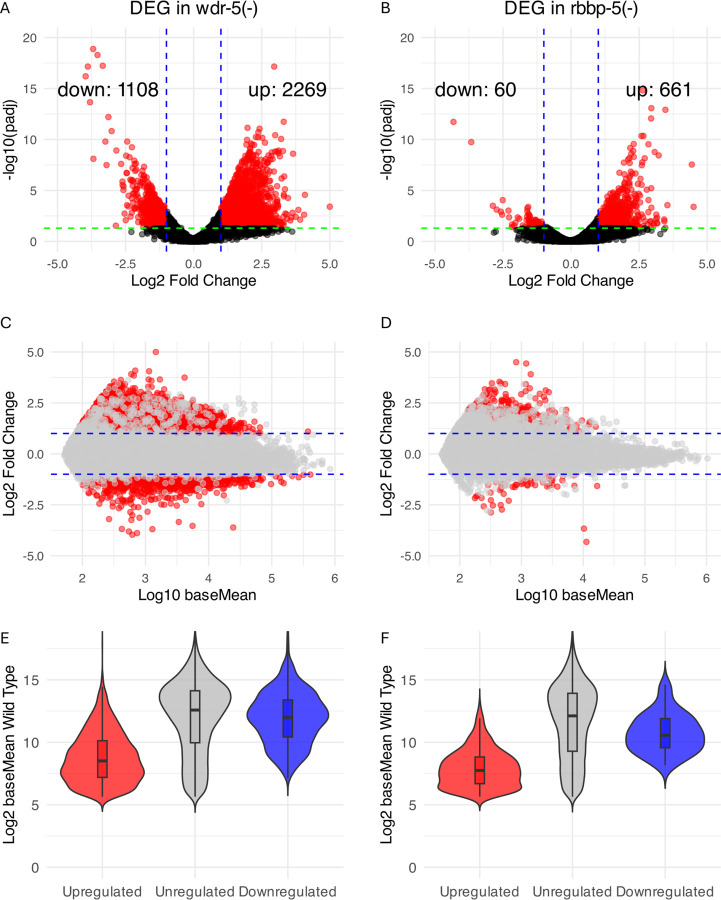
WDR-5 impacts the transcriptome more profoundly than RBBP-5. (**A** and **B**) Volcano plots showing differentially expressed genes (DEG) in *wdr-5(-)* and *rbbp-5(-)* embryos compared to wild-type. DEG (red dots) were defined as having >2-fold change and adjusted p < 0.05. Loss of WDR-5 results in widespread dysregulation (2269 upregulated, 1108 downregulated genes), while RBBP-5 loss leads to a more limited response (661 upregulated, 60 downregulated genes). (**C** and **D**) MA plots showing DEG (red) plotted against log10 baseMean expression. Data distribute symmetrically along the y-axis zero line, indicating no systemic bias introduced by DESeq2 modelling. (**E** and**F**) Violin and box plots showing baseline (wild-type) expression levels for up-regulated, down-regulated, and un-regulated genes in each mutant. Down-regulated genes tend to have higher baseline expression in wild type than up- or un-regulated genes. Up-regulated genes tend to have a lower baseline expression in wild type than down- or un-regulated genes. All three groups for both mutants are statistically different (p< 0.01) using the Mann-Whitney U test.

In *wdr-5(-)* embryos, we identified 3377 DEG, comprising 1108 down-regulated genes and 2269 up-regulated genes (Fig. 7A, Table S1). In contrast, the *rbbp-5(-)* mutant exhibited only 721 DEGs, with 60 down-regulated and 661 up-regulated (Fig. 7B, Table S1). The predominance of up-regulated genes was to an extent unexpected, given the association of H3K4 methylation with active transcription, though previous studies have also noted this prevalence. Our findings are therefore consistent with prior transcriptomic analyses comparing *wdr-5(-)* and *set-2(-)* mutants in dissected *C. elegans* gonads. The authors similarly reported a bias towards up-regulation [44]. However, another study using *set-2* mutants in early embryos reported a more balanced distribution of down- and up-regulated genes [32].

Upon re-analysing this early embryonic dataset [32] with both a two-fold change cutoff and padj < 0.05, we found an enrichment for up-regulated genes (587 up versus 203 down; Table S2), which is in line with our findings. To assess the performance of DESeq2 normalisation, we generated MA plots for each comparison (Fig. 7C, D). Data points were centred around log2 fold change = 0 at low expression levels, confirming successful normalisation. The data are also consistent with the effects observed on the *wdr-5(-)* and *rbbp-5(-)* respective transcriptomes, whereby more genes are up-regulated than down-regulated in both mutants.

We next asked whether down-regulated genes in the mutants tend to be highly expressed in wild type, given the known association between highly expressed genes and H3K4 methylation. To assess this point, we separated the data into down-, up-, and un-regulated genes and plotted their associated levels of expression in wild type (baseMean wild type) for each corresponding mutant (Fig. 7E and F). Indeed, down-regulated genes in each mutant exhibited significantly higher expression in wild type than up-regulated genes and un-regulated genes (pval < 0.01), suggesting that these down-regulated genes are more likely to represent direct WDR-5 or RBBP-5 targets (Fig. 7E, F).

Finally, to assess the global impact of WDR-5 and RBBP-5 loss on their respective transcriptomes, we quantified the net directional change in expression across all DEG. This metric integrates both the direction (log2 fold change) and magnitude (baseMean) of expression changes to summarise transcriptomic shifts. In *wdr-5(-)* embryos, we observed a net change of −28%, indicating an overall decrease in gene expression among mis-regulated genes (Table S3). In contrast, *rbbp-5(-)* embryos showed a milder net change of −9% (Table S3). Thus, while both mutants exhibit more up- than down-regulated genes, the overall net effect is a reduction in number of transcripts. This effect is more pronounced in *wdr-5(-)*, consistent with its broader transcriptomic perturbation (Fig. 7A and B).

### WDR-5 exhibits H3K4 methylation-independent functions impacting on gene expression

**Figure 8:**
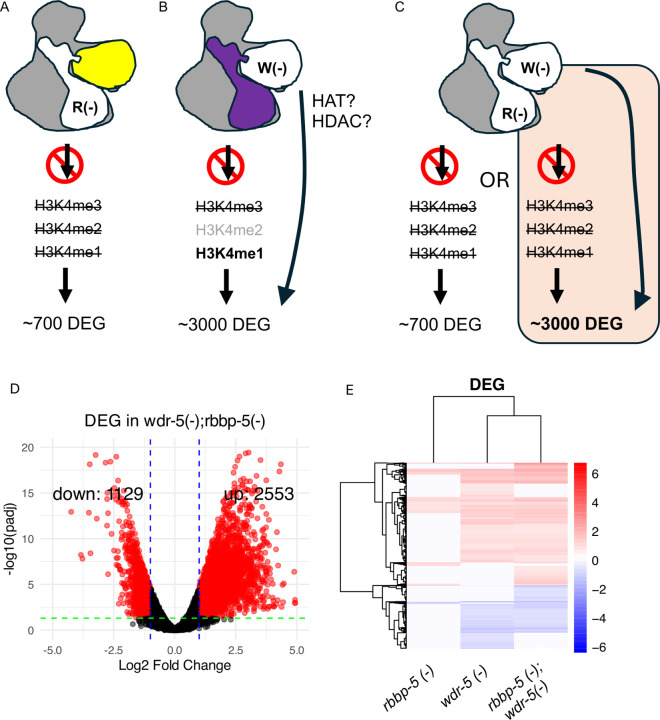
WDR-5 regulates gene expression independently of H3K4 methylation. (**A-C**) Experimental rationale for generating an *rbbp-5(-)*; *wdr-5(-)* double mutant to assess whether transcriptional changes in *wdr-5(-)* are due to persistent H3K4 methylation. **(A)** In the absence of RBBP-5, H3K4 methylation is abolished. **(B)** In the absence of WDR-5, H3K4me2 is erroneously distributed but still present whereas H3K4me1 levels are increased. Parallel activities associated with Histone acetylation and deacetylation (HAT and HDAC) are depicted by the side line. (C) Two hypotheses: if WDR-5 acts through H3K4 methylation only, the double mutant should resemble *rbbp-5(-)*; if WDR-5 also has parallel activities independent of H3K4 methylation, the double mutant should resemble *wdr-5(-)*. The double mutant embryos lacking both RBBP-5 and WDR-5 show transcriptomic deregulation similar to the single *wdr-5(-)* mutant, thus favouring the model whereby H3K4 methylation independent activities are implicated. **(D)** Volcano plot shows DEG in *rbbp-5(-)*; *wdr-5(-)* embryos relative to wild type. There are 2553 significantly up-regulated genes versus 1129 down-regulated genes. **(E)** Hierarchical clustering reveals that the transcriptomes of the *wdr-5(-)* single mutant and the *rbbp-5(-)*; *wdr-5(-)* double mutants are highly similar. Heatmap shows z-score normalised expression values for DEG across the *rbbp-5(-)*and *wdr-5(-)* single mutants and the double *rbbp-5(-)*; *wdr-5(-)* mutant. Genes are clustered by expression pattern across the three genotypes.

We next asked the question whether the large number of DEG in *wdr-5(-)* could be attributed to the persistence of H3K4me1, H3K4me2 or residual H3K4me3. To test this, we generated an *rbbp-5(-)*; *wdr-5(-)* double mutant, in which H3K4 methylation is unlikely because of the absence of RBBP-5. We made two predictions as to how the *rbbp-5(-)*; *wdr-5(-)* double mutant would affect transcription (Fig. 8A–C). One is that the transcriptomic profile of the double mutant will resemble that of *rbbp-5(-)* alone (~ 700 DEG) because the perturbations in the *wdr-5(-)* transcriptome are explained by persistent H3K4me1, H3K4me2 or residual H3K4me3 (Fig. 8C; left panel). The second possibility is that the transcriptomic profile of the double mutant will mirror the *wdr-5(-)* transcriptome (~ 3000 DEG) because the absence of RBBP-5 does not interfere with WDR-5 parallel activity (Fig. 8C; right panel). It turns out that the *rbbp-5(-)*; *wdr-5(-)* double mutant exhibited 3682 DEG (Fig. 8D), a number comparable to the *wdr-5(-)* single mutant (3377 DEGs; Fig. 7A). Clustering analysis (Fig. 8E) and Venn diagrams (Suppl. Figure 3) confirmed substantial overlap between the DEG of the double and *wdr-5(-)* mutants. Moreover, a large proportion of the DEG identified in *rbbp-5(-)* were also found in *wdr-5(-)* and in the double mutant, consistent with both proteins acting within the SET/MLL complex (Suppl. Figure 3). However, the broader transcriptional changes observed in the absence of WDR-5 (even when H3K4 methylation is abolished) strongly suggest that WDR-5 has additional functions beyond promoting H3K4 methylation.

**Figure 9:**
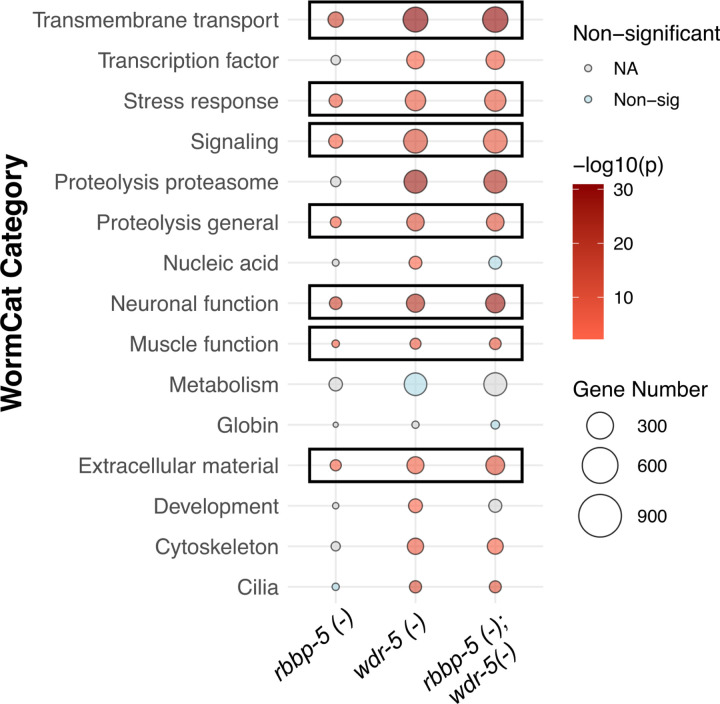
Most functional categories of DEG are shared between the single mutants and the *rbbp-5(-)*; *wdr-5(-)* double mutant. Bubble plot shows the distribution of DEG across WormCat2.0 functional categories for each genotype. Circle size corresponds to the number of DEG per category and colour reflects enrichment score as indicated. The outlined categories are shared across all three geneotypes.

We next compared Gene Ontology categories between the three datasets using WormCat 2.0 [59] and found that many of the categories are shared between the single *wdr-5(-)* and *rbbp-5(-)* mutants and the double *rbbp-5(-)*; *wdr-5(-)* mutant, indicating that those functions such as neuronal and stress response are likely regulated, at least in part, by H3K4 methylation (Fig. 9). Other WormCat categories (transcription factors, cytoskeleton, and cilia) are shared between the single *wdr-5(-)* mutant and the double *rbbp-5(-)*; *wdr-5(-)* mutant and these are likely regulated by WDR-5 parallel activity. Taken together, these data show that the transcriptomes of the single *wdr-5(-)* and the double *rbbp-5(-)*; *wdr-5(-)* mutants share a high degree of similarity, indicating that WDR-5 can affect gene expression independently of H3K4 methylation during *C. elegans* embryogenesis.

## Discussion

Our study shows that WDR-5 and RBBP-5 have distinctive roles in the deposition of methyl marks at H3K4. WDR-5 facilitates H3K4 tri-methylation, whilst RBBP-5 is necessary for mono- and multi-methylation. These functions align with cryo-EM structural studies showing that RBBP5 plays a central role in nucleating the MLL1 core complex on the nucleosome, and engaging both its DNA and histone surfaces to orient the complex [21, 60–62]. Our data are also largely consistent with other studies performed on *C. elegans* embryos [35, 36, 63]. We also show that WDR-5 has intricate and more subtle functions; its absence reduces H3K4 methylation more prominently on autosomes than on the sex chromosome and the effects on deposition of H3K4me2 can be either positive or negative depending on the genomic features involved. Crucially, we were able to functionally demonstrate that WDR-5 exhibits H3K4 methylation-independent activity by analysing the transcriptome of a double mutant between *rbbp-5(-)* and *wdr-5(-)* and comparing it with the single mutants.

Our study shows that WDR-5 has profound impacts on gene expression that are independent of its role in H3K4 methylation. WDR-5 is found physically associated with additional chromatin complexes. These ‘moonlighting’ activities are likely explaining our results whereby the alterations in the transcriptome of the *wdr-5(-)* mutant are more profound than in the *rbbp-5(-)* transcriptome. This conclusion is based on our results from the double *rbbp-5(-)*; *wdr-5(-)* mutant showing that most of these *wdr-5(-)* alterations in gene expression are independent of H3K4 methylation, since H3K4 methylation is abrogated without RBBP-5. Thus, our work supports the proposal by Guarnaccia et al. that WDR-5 acts as a multi-functional hub in the nucleus [25].

WDR5 was first characterised in mammals as a core component of the H3K4 methylation complex [64]. However, it was later found in other complexes notably the NSL (Non-Specific Lethal) and ATAC (Ada Two A Containing) histone acetyltransferase complexes [28, 65]. WDR5 can also be found in histone deacetylases complexes such as RPD3 HDAC in yeast (or mSin3a-HDAC1 in mammals) and the NuRD complex [31]. ING2, a component of the sSin3a-HDAC1, interacts via its PHD domain with H3K4me3 to stimulate deacetylation and repress transcription [66]. In yeast, it was found that H3K4me2/me3 can recruit RPD3 HDAC to repress cryptic transcription [14]. In *C. elegans*, WDR-5 is found within the Sin3S HDAC repressive complex [32], which is similar to RPD3 HDAC in yeast and sSin3a-HDAC1 in mammals. Interestingly, work in other species support an H3K4 methylation-independent role for WDR5. WDR5 point mutations defective in H3K4 methylation could still rescue specific phenotypes such as mouse embryonic stem cells self-renewal defects and de-repression of germ cell specific genes [67] as well as left-right patterning of the heart in Xenopus [68], indicating that H3K4 methylation-independent activity is conserved in vertebrates. Thus, WDR-5 appears to act as a multi-functional hub regulating both activation and context-dependent repression of transcription via H3K4 methylation as well as histone acetylation and deacetylation. This promiscuous activity most likely explains the effects on the *wdr-5(-)* transcriptome reported herein. However, whether and how any of these activities are privileged over others remains to be elucidated.

In contrast to WDR-5, RBBP-5 is critical and specific to the deposition of H3K4 methyl groups. Concordant with our work, a study investigating crosstalk between the NSL and SET/MLL complexes in *Drosophila* has shown that depletion of RBBP5 affects H3K4me2 deposition but not H4K16 acetylation, indicating that RBBP5 is not directly affecting histone acetylation, whereas WDR5 inactivation can affect both H3K4 methylation and H4K16 acetylation [29]. In addition, a *C. elegans* study investigating masculinisation of the germline found that this phenotype arises in the absence of WDR-5 but not in the absence of RBBP-5, indicating that WDR-5 has H3K4 methylation-independent activity but not RBBP-5 [69]. It is also interesting to note that loss-of-function mutations in RBBP-5 in humans have recently been identified and associated with neurodevelopmental disorder, short stature and microcephaly [70], highlighting the importance of H3K4 methylation for neuronal function.

Our analysis has also revealed a chromosomal hierarchy in H3K4me3 enrichment at transcription start sites, which closely mirrors the pattern of phenotypic enrichment observed through systematic RNAi screening [71]. Specifically, chromosomes I and III exhibit the highest levels of H3K4me3, followed by chromosomes II and IV, and with chromosomes V and X showing the lowest enrichment. This pattern is consistent with previous findings showing that chromosomes I and III are relatively enriched for active chromatin marks, including H3K4 methylation, whereas chromosomes V and X are comparatively depleted [47]. One possible explanation linking H3K4me3 enrichment to RNAi phenotypic outcomes is that genes which are robustly transcribed (and therefore more heavily marked by H3K4me3) are more likely to yield observable phenotypes when knocked down by RNAi. Although the mechanisms coordinating transcriptional regulation at the chromosome-wide level remain poorly understood, it is plausible that differential H3K4me3 enrichment reflects the partitioning of chromosomes into distinct chromatin environments during early embryogenesis. Higher levels of H3K4me3 on chromosomes I and III may indicate a more open, transcriptionally permissive architecture, possibly maintained through large-scale domain organisation or preferential spatial positioning within the nucleus. Conversely, the lower H3K4me3 levels on chromosomes V and X could reflect sequestration into less active chromatin territories. Notably, data from a recent *C. elegans* study investigating partitioning of chromatin states in the germlines has also revealed that transcriptionally active domains (marked by high H3K4me3 and H3K36me3) follow a similar chromosomal hierarchy [72]. These chromosome-wide biases in chromatin accessibility and transcriptional competence may underlie the observed correspondence between H3K4me3 and RNAi phenotype enrichment.

Our chromosome-level analysis also shows that H3K4me1 enrichment and distribution at TSS on chromosome X are distinct from those on autosomes. It is therefore tempting to speculate that H3K4me1 could play a role on chromosome X to dampen transcription. Alternatively, as shown during the early stages of X chromosome inactivation in mammals [73], elevated H3K4me1 levels may reflect reduced transcription and the incomplete conversion into H3K4me2 and H3K4me3. There is also the possibility that the SET/MLL complex could play a role in regulating the dosage compensation (DC) complex. It has been shown that DPY-30, an additional scaffolding component of the SET/MLL complex, is also present in the DC complex [74]. The authors show that DPY-30 and ASH-2 are found at a subset of sites known to be critical for dosage compensation. Since ASH-2 knockdown does not affect the recruitment and function of the DC complex, it seems that the SET/MLL complex would act downstream or in parallel. It is plausible that the co-localisation of the DC and SET/MLL complexes at the same DC binding sites prevents enrichment of H3K4me3 and thereby increases the levels of H3K4me1, which would be consistent with our findings.

## Conclusion

Together, our findings establish that WDR-5 and RBBP-5 exert distinct functions in chromatin regulation, with WDR-5 having a broader influence on gene expression through mechanisms most likely beyond H3K4 methylation. This highlights WDR-5 as a versatile regulatory scaffold in chromatin biology. Future work will be needed to determine how WDR-5’s roles across different chromatin-modifying complexes are coordinated, and whether specific chromatin contexts favour particular functions over others. Defining the precise interactome of WDR-5 *in vivo* will help clarify these mechanisms and further understanding of its roles in development and disease.

## Supplementary Files

This is a list of supplementary files associated with this preprint. Click to download.

- SamsudinTableS1.xlsx
- SamsudinTableS2.xlsx
- SamsudinTableS3.xlsx
- SamsudinSupplfigure1.pdf
- SamsudinSupplfigure2.pdf
- SamsudinSupplfigure3.pdf

## Data Availability

The ChIP-seq data have been deposited in the GEO repository under ID code GSE94639 and RNA-seq data have been deposited in the ArrayExpress repository under ID code E-MTAB-15080.
